# Exploring the Functions of Mutant p53 through *TP53* Knockout in HaCaT Keratinocytes

**DOI:** 10.3390/cimb46020094

**Published:** 2024-02-08

**Authors:** Daniil Romashin, Alexander Rusanov, Viktoriia Arzumanian, Alexandra Varshaver, Ekaterina Poverennaya, Igor Vakhrushev, Alexander Netrusov, Nataliya Luzgina

**Affiliations:** 1 Institute of Biomedical Chemistry, Moscow 119121, Russia; dromashin@ibmc.msk.ru (D.R.); viktoriia.arzumanian@ibmc.msk.ru (V.A.); 4varshaver@gmail.com (A.V.); k.poverennaya@ibmc.msk.ru (E.P.); vakhrunya@gmail.com (I.V.); ng-luzgina@mail.ru (N.L.); 2 Faculty of Biology, Lomonosov Moscow State University, Moscow 119234, Russia; anetrusov@mail.ru; 3 Faculty of Biology and Biotechnology, HSE University, Moscow 101000, Russia

**Keywords:** *TP53*, p53, HaCaT, transcriptomics, gene knockout

## Abstract

Approximately 50% of tumors carry mutations in *TP53*; thus, evaluation of the features of mutant p53 is crucial to understanding the mechanisms underlying cell transformation and tumor progression. HaCaT keratinocytes represent a valuable model for research in this area since they are considered normal, although they bear two gain-of-function mutations in *TP53*. In the present study, transcriptomic and proteomic profiling were employed to examine the functions of mutant p53 and to investigate the impact of its complete abolishment. Our findings indicate that CRISPR-mediated *TP53* knockout results in significant changes at the transcriptomic and proteomic levels. The knockout of *TP53* significantly increased the migration rate and altered the expression of genes associated with invasion, migration, and EMT but suppressed the epidermal differentiation program. These outcomes suggest that, despite being dysfunctional, p53 may still possess oncosuppressive functions. However, despite being considered normal keratinocytes, HaCaT cells exhibit oncogenic properties.

## 1. Introduction

The transcription factor p53 is widely known as a tumor suppressor and ‘the guardian of the genome’ [1]. Mutations in *TP53*, resulting in the loss of oncosuppressive functions of the encoded protein p53, represent an important stage in cancer progression, which applies to tumors originating from keratinocytes [2]. This, in turn, may lead to the accumulation of other oncogenic mutations in genes such as *NOTCH2*, *EGFR,* and *PIK3CA* [3].

Mutations in *TP53* are found in approximately 50% of skin cancer cases; thus, investigation of the features of mutant p53 is crucial for understanding the mechanisms underlying cell transformation and tumor progression [4]. HaCaT keratinocytes, a line of spontaneously immortalized human keratinocytes, represent a convenient model for research in this field [5]. Although HaCaT cells are traditionally defined as normal non-tumoral keratinocytes and are commonly used as an alternative to primary keratinocytes [6,7,8], they also bear two UV-signature gain-of-function (GOF) mutations in both alleles of *TP53* (R282Q and H179Y) [9]. The novel properties of p53 obtained through mutations are manifested in its capacity to bind atypical response elements and its altered affinity to other transcription factors, which in turn results in the suppression of their biological functions [10]. Although two earlier studies involved functional inactivation of p53 (shRNA-mediated knockdown), both studies focused on investigating specific properties of p53, such as its interaction and cooperation with other transcription factors including p63 and NF-kB B [10,11]. It has been reported that mutant p53 in cancer cells can suppress different targets of normal p53, whereas inactivation of mutant p53 in these cells results in cell cycle arrest and apoptosis induction [12]. In contrast, several studies have indicated that mutant p53 in HaCaT cells is at least partially functional, as it induces apoptosis and cell cycle arrest in UV-irradiated HaCaT cells [13,14]. Nonetheless, many features of mutant p53 in HaCaT cells remain unclear.

The presence of mutant p53 is not the only feature distinguishing HaCaT cells from primary keratinocytes. HaCaT cells are characterized by aberrant NF-kB activity, which is associated with increased sensitivity to UVB-induced apoptosis [15]. Moreover, after chronic exposure to UVB, HaCaT cells exhibit properties of an aggressive form of skin cancer, such as altered proliferation and migration rate and induction of the epithelial–mesenchymal transition (EMT) [16]. Consequently, it would be erroneous to assume that the abolishment of mutant p53 would eliminate the differences between HaCaT cells and primary keratinocytes. However, HaCaT cells are of interest in the context of the biology of keratinocytes bearing UV-induced mutations in *TP53.*

Functional inactivation of *TP53* is a powerful tool for investigating the features of mutant p53, its functional status, and its properties. In addition, the functional characteristics of p53-deficient HaCaT may provide new data regarding HaCaT biology and their resemblance to that of normal and transformed cells. Previously, we generated a stable HaCaT cell line with CRISPR-inactivated *TP53* [17]. The present study was based on a complex analysis of omics data obtained from *TP53*-KO HaCaT cells and aimed to elucidate the functions and properties of mutant p53 in HaCaT keratinocytes.

## 2. Materials and Methods

### 2.1. Cell Culture and Treatment

HaCaT cells were purchased from DKFZ Collection (CLS Cell Lines Service, 300493, Eppelheim, Germany). *TP53*-KO HaCaT cells were generated using the CRISPR/Cas system as previously described [17]. Employing paired Cas9n D10A mutant nucleases resulted in multiple insertions and deletions in the alleged *TP53* region (positions 7676184–7676212).

The cells were cultured in DMEM/F12 (1:1) medium containing 10% fetal bovine serum, penicillin/streptomycin (100 U/mL and 100 μg/mL), and 1% GlutaMAX^TM^ (all from Gibco, Billings, MT, USA). Cells were cultured in 25 cm^2^ tissue-treated flasks or 60 mm Petri dishes (Corning, New York, NY, USA), and the culture medium was replaced every other day. For gene expression evaluation, cells were seeded in 6-well culture plates at a density of 3.0 × 10^5^ cells per well (Corning, NY, USA) and grown until they reached full confluence (48 h). The medium was then replaced with fresh medium containing calcium chloride (2.8 mM) and the cells were cultured for 9 days. For culture at the air–liquid interface, the cells were seeded into 24-well plates with cell culture inserts (Wuxi NEST Biotechnology, Wuxi, China) at a density of 5.0 × 10^5^ cells per insert. Regular culture medium was supplemented with calcium chloride (2.8 mM), ascorbic acid (50 µg/mL), and 2 ng/mL keratinocyte growth factor (Gibco, Billings, MT, USA). The cells were cultured submerged for 48 h, after which the medium was discarded from the inserts. The cells were cultured at the air–liquid interface for 14 days. The culture medium was replaced daily with a fresh medium.

### 2.2. Transcriptomic Analysis

For transcriptomic analysis, HaCaT cells were seeded onto 100 mm Petri dishes (Corning, NY, USA) at a density of 1.0 × 10^6^ cells per plate. After 24 h, the cells were collected by trypsinization followed by RNA isolation. Total RNA was isolated according to the manufacturer’s protocol and quantified using a NanoDrop-1000 spectrophotometer (Thermo Fisher Scientific, Waltham, MA, USA). Transcriptome data were obtained by high-throughput paired-end sequencing using an Illumina NovaSeq 6000 with a read length of 100 bp. The TruSeq Stranded mRNA Library Prep Kit was used to prepare the RNA libraries. All steps were performed in accordance with the manufacturer’s protocol. Transcriptome profiling was performed in triplicate with a separate process of RNA extraction for each replicate. Raw sequencing data were uploaded to the NCBI SRA (https://www.ncbi.nlm.nih.gov, accessed on 30 November 2023). The accession number is PRJNA1005459 for both WT and *TP53*-KO cells.

### 2.3. Proteomic Analysis

Previously published proteomic data have been used to identify differentially expressed proteins between cell lines. Proteomic profiling is described in detail in the original data descriptor paper [17]. Briefly, cell pellets were lysed in lysis buffer, and protein precipitation was performed using the methanol–chloroform method. Protein extracts were prepared for MS analysis by diluting in denaturation buffer and adding trypsin for in-solution digestion. Peptides were separated via HPLC and analyzed using LC-MS/MS. Raw proteomic data were deposited to the ProteomeXchange Consortium via the PRIDE partner repository (http://www.proteomexchange.org/Project, accessed on 19 January 2024, accession: PXD030700).

### 2.4. Bioinformatic Analysis

To ensure the quality of the fastq files, we employed FastQC [18]. Salmon [19] was used to quantify the expression in transcripts per million units (TPMs). Gene expression was calculated by aggregating all TPMs for the corresponding transcripts. The identification was performed against a concatenated target/decoy version of the Homo sapiens complement of the UniProtKB (July 2021). Raw proteome data were processed according to the protocol described earlier [17]. Concisely, we used MaxQuant software (version 1.6.3.4) for label-free quantitation.

The R software environment was used for the computation and visualization of transcriptomic and proteomic data (ver. 4.1) (R: The R Project for Statistical Computing https://www.r-project.org/, accessed on 8 August 2023). We used the DESeq2 package [20] for differential gene expression analysis. For visualization, we used EnhancedVolcano for the volcano plot (https://github.com/kevinblighe/EnhancedVolcano, accessed on 25 July 2023) and ClusterProfiler for geneset enrichment analysis [21] as described in Poverennaya et al. [22]. As a source of biological knowledge for transcriptomic data, we utilized the Wikipathways [23] and PROGENy databases [24]. We used a previously published dataset to count differential protein expression [17]. To enrich differentially expressed proteins in our proteomic results with biological knowledge, we leveraged Gene Ontology (GO), specifically focusing on molecular function (MF) orthogonal ontology [25]. The general EMT score was estimated based on gene expression signatures. The computation was performed using a two-sample Kolmogorov–Smirnov test [26].

### 2.5. RT-qPCR

Total RNA was isolated using the RNeasy Kit (QIAGEN, Venlo, The Netherlands), according to the manufacturer’s instructions. cDNA synthesis was performed using the MMLV reverse-transcription kit (Evrogen, Moscow, Russia) utilizing 1 µg of RNA per reaction. SYBR label (Evrogen, Russia) was used for quantitative PCR in the Bio-rad CFX connect system. Relative gene expression was calculated with the ΔΔCt method with *GAPDH* and *ACTB* as reference genes [27]. The results of two biological and three technical replicates were used for the analysis and are presented as the mean ± SEM. Raw data were analyzed using the CFX Maestro 1.0 software (v. 4.0.0325.0418). The stability of housekeeping genes employed for normalization was evaluated using CFX Maestro 1.0 software (v. 4.0.0325.0418). The primer sequences used for the analysis are listed in Table 1.

### 2.6. Migration Assay

An Incucyte^®^ ZOOM System (Sartorius, Göttingen, Germany) was used for the scratch test. WT and *TP53*-KO cells were seeded in an Incucyte^®^ Imagelock 96-well plate at a density of 2.0 × 10^6^ cells per well and grown until they reached a full monolayer (24 h). To suppress proliferation, the cells were pretreated with mitomycin C. In brief, 2 h prior to wounding, the culture medium was replaced with serum-free medium containing 10 µg/mL mitomycin C (Merck, Rahway, NJ, USA). The injury was inflicted using an IncuCyte 96-pin wound-making tool (Essen Bioscience, Göttingen, Germany). Once the injury was inflicted, cells were rinsed twice with fresh medium and cultured for 24 h. Images were captured hourly. The wound area was calculated using Incucyte^®^ ZOOM Software (v. 2016A) (Sartorius, Germany).

### 2.7. Confocal Fluorescent Microscopy

The culture inserts were fixed in 10% buffered formaldehyde, permeabilized with 0.1% Triton X-100, and blocked with 1% BSA in PBS-Tween 20^TM^ for 30 min under rotation (room temperature). A keratin 1 (CK1) primary antibody was used for detection and a secondary Alexa 488-conjugated antibody was used for visualization (Finetest, Hubei, China). Hoechst 33342 (Thermo Fisher Scientific, USA) was used for nuclei visualization. Images were obtained using an inverted confocal laser scanning microscope Zeiss LSM 880 with AiryScan, a 32-channel GaAsP-PMT area detector, a Plan-Apochromat 40×/1.3 Oil DIC M27 (WD = 0.2 mm), and a (UV) VIS-IR objective with Scan zoom 2. A Z-stack of a 27–30 µm depth was scanned for one field of view in two randomly chosen locations for each sample with a 0.1 µm × 0.1 µm pixel size and a 106.27 µm × 106.27 µm image size in AiryScan SR mode with a 2.05 µs pixel time. AiryScan processing, Z-stack maximal intensity projection, and histogram Min/Max correction were performed using the Zeiss ZEN 2.3. Zeiss LSM 880 with Airyscan is equipment belonging to the Core Centrum of the Institute of Developmental Biology RAS, Moscow, Russia.

### 2.8. Proliferation Assay

For cell proliferation evaluation, a CytoTrace™ Red fluorescent probe was used (AAT Bioquest, Pleasanton, CA, USA). The cells were seeded in 100 mm Petri dishes at a density of 3.0 × 10^5^ (Corning, USA) and cultured as described in Section 2.1 for 24 h. The day after seeding, the culture medium was replaced with DMEM/F12 serum-free medium containing fluorescent dye for 30 min. After incubation, the medium was replaced with a regular culture medium. Cells were cultured for 48 h, harvested by trypsinization, and fixed in 4% buffered formaldehyde. The control cells (day 0) were collected immediately after incubation with the fluorescent probe. Fluorescence was analyzed with a ZE5 flow cytometer (Bio-rad, Hercules, CA, USA). The raw data were analyzed using Floreada.io software (https://floreada.io/, assessed on 10 November 2023). The graph represents the median fluorescence intensity (MFI) delta between the control cells and the cells collected 48 h post-seeding.

### 2.9. Flow Cytometry

For flow cytometry analysis, the cells were cultured under standard conditions in 6-well culture plates (Corning, USA), as described in Section 2.1. Cells were harvested by trypsinization and rinsed twice with HBSS (Capricorn Scientific, Ebsdorfergrund, Germany). After washing, the cells were resuspended in FACS Buffer (DPBS without Ca^2+^/Mg^2+^, 2 mM EDTA, 1% FBS). An APC-conjugated anti-human PD-L1 antibody (Sony Biotechnology, San Jose, CA, USA) was used for PD-L1 detection. An APC-conjugated anti-human CD324 antibody (Miltenyi Biotec, Bergisch Gladbach, Germany) was used for CD324 staining. For CD325 staining, an APC-conjugated anti-human CD325 antibody was used (BioLegend, San Jose, CA, USA). The cells were stained in the dark for 15 min at RT. The samples were analyzed using a NovoCyte Flow Cytometer (Acea Biosciences, San Diego, CA, USA). Novo Express software (v. 1.2.4) was used for raw data analysis. The stain index (SI) was calculated using the following formula: (MFI of positive peak—MFI of negative peak)/2 × standard deviation of negative peak.

### 2.10. Statistical Analysis

Proteomic and transcriptomic profiling, migration assays, and ICC were conducted in at least 3 biological replicates. For RT-qPCR, there were two independent biological replicates, each performed in triplicate. The graphs represent experimental data as means ± SEMs. Statistical analysis was carried out using one-way ANOVA with Tukey’s post hoc test. GraphPad Prism Version 8.0 software was employed for graph design (GraphPad Software LLC, San Diego, CA, USA).

## 3. Results

### 3.1. Transcriptomic Differences

**Figure 1:**
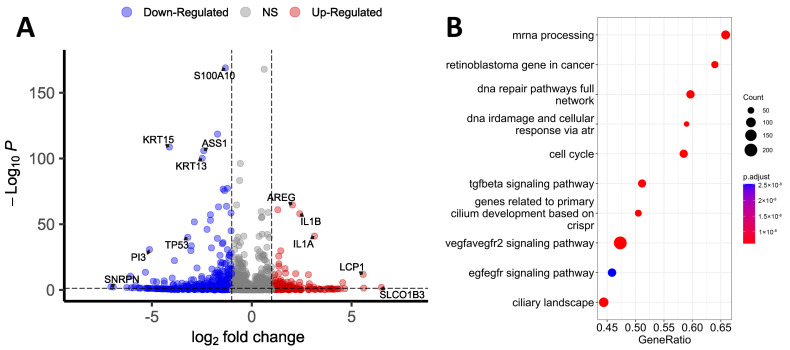
Transcriptomic profiling of WT and *TP53*-KO HaCaT cells. (**A**) Volcano plot showing differentially expressed genes between WT and *TP53*-KO cells. (**B**) Gene Set Enrichment Analysis of differentially expressed genes between WT and *TP53*-KO cells.

RNA-seq was primarily performed within the framework of omics profiling. Transcriptomic analysis identified 12 825 and 12 368 genes in wild-type (WT) and *TP53*-KO cells, respectively (TPM, transcripts per million > 0). Differential expression analysis revealed 395 genes with altered expression (Figure 1A, Supplementary Table S1). Among the genes with altered expression, 86 genes were upregulated and 309 genes were downregulated in *TP53*-KO cells compared with WT. Genes with differential expression were sorted in decreasing order of log_2_ fold change (log_2_FC) and filtered according to the criterion of discovery rate (FDR < 0.05).

We conducted a Gene Set Enrichment Analysis (GSEA) of filtered genes to identify the pathways affected by the knockout of *TP53* (Figure 1B). According to the results, the most enriched pathways were ‘mRNA processing’ and ‘retinoblastoma gene in cancer’. Pathways like ‘DNA repair pathways full network’, ‘DNA IRdamage’, ‘cellular response via atr’, and ‘cell cycle’ correspond to the established role of p53 in the regulation of cell cycle control and DNA reparation [1]. The enrichment of pathways like ‘genes related to primary cilium development’ and ‘ciliary landscape’ was unanticipated, but these outcomes may provide a basis for further investigation since altered ciliation is common for patients with atopic dermatitis and psoriasis, and HaCaT cells represent a convenient model for the investigation of ciliation [28].

Among the genes with altered expression, *SLCO1B3* had the highest log_2_FC (log_2_FC = 6.49). This gene encodes transmembrane receptor H, which facilitates the intracellular transport of a broad range of organic anions [29,30]. A high log_2_FC was also observed for *LCP-1* (log_2_FC = 5.57), which encodes plastin-2. This protein activates cell migration and invasion, and its altered expression correlates with poor prognosis in cases of lung squamous cell carcinoma [31]. *ISM1* was upregulated (log_2_FC = 3.97) in knockout cells. This gene is known for its functions in the regulation of the immune response and the suppression of angiogenesis [32]. Several cytokine-coding genes were upregulated in *TP53*-KO cells, including *IL1A* (log_2_FC = 3.14) and *IL1B* (log_2_FC = 2.4). In addition, *IL1R1* was upregulated (log_2_FC = 4.55). *CD274* (PD-L1) expression was markedly increased in *TP53*-KO cells (log_2_FC= 3.10). Altered expression of PD-L1 may be associated with the evasion of the immune response and is common for many tumors [33]. PD-L1 is also an important mediator of the EMT [34]. *AREG* expression was also markedly increased (log_2_FC = 2.03). This gene is associated with a psoriasis-like skin phenotype [35] and induces the EMT in pancreatic cancer cells through EGFR/ERK/NF-kB [36]. The protein encoded by this gene is an autocrine growth factor and mitogen for astrocytes, Schwann cells, and fibroblasts. This protein interacts with the EGF/TGF-α receptor to promote the growth of normal epithelial cells and inhibits the growth of some aggressive carcinoma cell lines. As a result of *TP53* knockout, *MMP13* expression significantly increased (log_2_FC = 2.57). This gene encodes a member of the M10 peptidase family of matrix metalloproteinases (MMPs). Proteins of this family are involved in the breakdown of the extracellular matrix in normal physiological processes, as well as in pathological processes, including metastasis [37,38]. In addition to *MMP13*, the expression of *TIMP2* (log_2_FC = −2.90), a tissue inhibitor of metalloproteinases, was markedly reduced in the *TP53*-knockout cells. The expression of various metalloproteinases and suppression of their inhibitors are not typical for epithelial cells and can be considered a marker of EMT [37]. As a result of knockout, *DLC-1* expression also increased (log_2_FC = 2.93). *DLC-1* encodes GTPase-activating protein (GAP) [39]. The GAP family of proteins is involved in the regulation of cytoskeletal changes. The *DLC-1* gene acts as a tumor suppressor gene in a number of common cancers, including prostate, lung, colorectal, and breast cancer [40]. Phosphorylated DLC-1 increases cell migration velocity but reduces directionality [41]. In addition, *LPXN* was upregulated in *TP53*-KO cells (log_2_FC = 2.10). The encoded protein leupaxin regulates migration and cell adhesion [42].

The expression of *MAGED1* was significantly decreased in *TP53*-KO cells (log_2_FC = −6.93). *MAGED1* is ubiquitously expressed and plays a role in anti-tumorigenesis in various cell types. *MAGED1* repression is a characteristic of tumor cells. Likewise, the expression of *FBP1*, which acts as a rate-limiting enzyme in gluconeogenesis, is commonly reduced in tumor cells [43]. In *TP53*-KO cells, *FBP1* expression was markedly reduced (log2FC = −5.32). Notably, in gastric cancer, a decrease in the level of this gene leads to EMT activation [44]. *CD9* expression was reduced (log_2_FC = −1.26) in *TP53*-KO cells. This gene plays an important role in the suppression of cancer cell motility and metastasis [45].

Several cytokeratin-coding genes decreased in *TP53*-KO cells. Specifically, *KRT4*, *KRT13*, *KRT15*, *KRT6B*, and *KRT16* were found to be downregulated (log_2_FC = −6.07, −2.47, −4.12, −3.11, −2.75, respectively). Keratin 13 (*KRT13*) dimerizes with type I keratin 4 and forms intermediate filaments that primarily line the cytoskeleton of specific epithelial cells. Reduced *KRT13* expression is observed in oral dysplasia, squamous cell carcinomas, and carcinomas in situ [46]. Keratin 13 expression is also decreased in TGF-β1-induced EMT [47]. In addition, in cells with *TP53* knockout, the expression of key markers of epidermal differentiation, *IVL* and *KRT10*, was noticeably reduced (log_2_FC = −1.25 and −3.87, respectively) [6,48,49].

**Figure 2:**
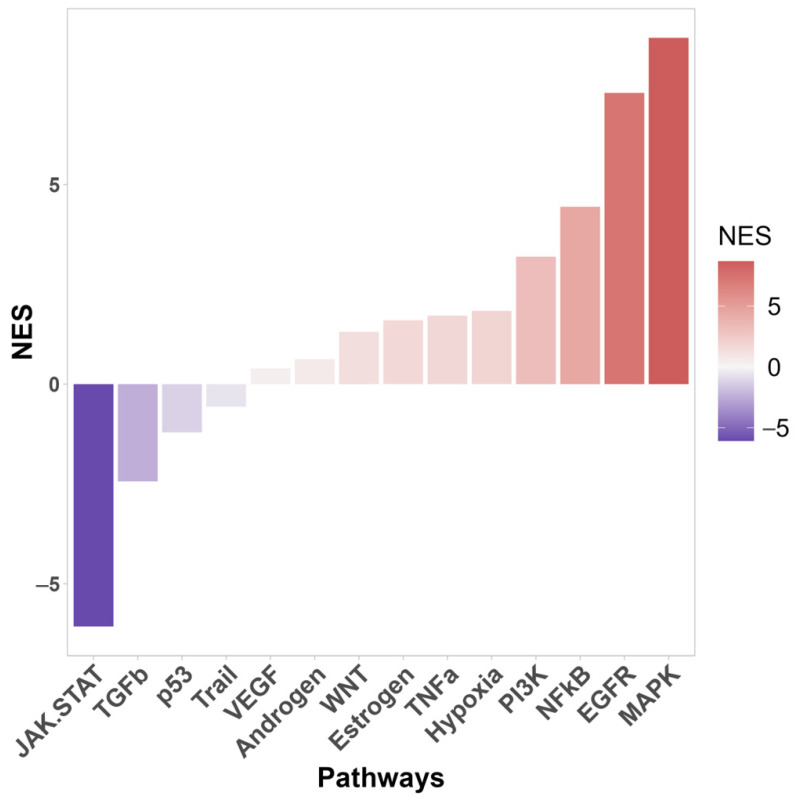
Signal pathway activity of differentially expressed genes between WT and *TP53*-KO cells using PROGENy database. NES—normalized enrichment score.

Enrichment analysis also identified the signaling pathways that were most significantly affected by *TP53* knockout (Figure 2). In knockout cells, activation of the MAPK, EGFR, NF-kB, PI3K, TNFa, and Wnt signaling pathways was most pronounced. The PI3K/AKT pathway is one of the most commonly activated signaling pathways in several human cancers, including melanoma, basal cell carcinoma (BCC), and squamous cell carcinoma (SCC) [50]. Hyperactivation of the MAPK pathway is important for cell transformation [51]. Wnt is activated upon wounding in the skin and is involved in each subsequent stage of the healing process [52].

The NF-kB signaling pathway plays a key role in maintaining skin homeostasis [53] and acts as a major transcriptional regulator of inflammation and immunity [54]. NF-kB is also as a key positive regulator of PD-L1 expression in cancer [55,56]. NF-κB directly induces the transcription of *CD274* by binding to its promoter and can also regulate PD-L1 at the post-transcriptional level [55].

### 3.2. Proteomic Differences

In the present study, we addressed previously published primary data obtained from proteomic analysis of WT and *TP53*-KO HaCaT cells to evaluate proteomic differences between cell lines [17]. In the aforementioned paper, we published the raw data obtained from both cell lines at a confluence of 50–60% (control) and after culture in a monolayer (full confluence) for 72 h. Samples were categorized based on the presence of mutations and confluency. In the present study, we focused on GO analysis of proteins with differential expression between lines in the subconfluent and full-confluent states. First, we analyzed the data of the control (subconfluent) group. As a result, 2644 proteins were identified in both cell lines. Among these proteins, 43 were significantly differentially expressed between the cell lines. The expression of five proteins was reduced in HaCaT cells after the *TP53* knockout, whereas thirty-eight proteins were upregulated.

**Figure 3:**
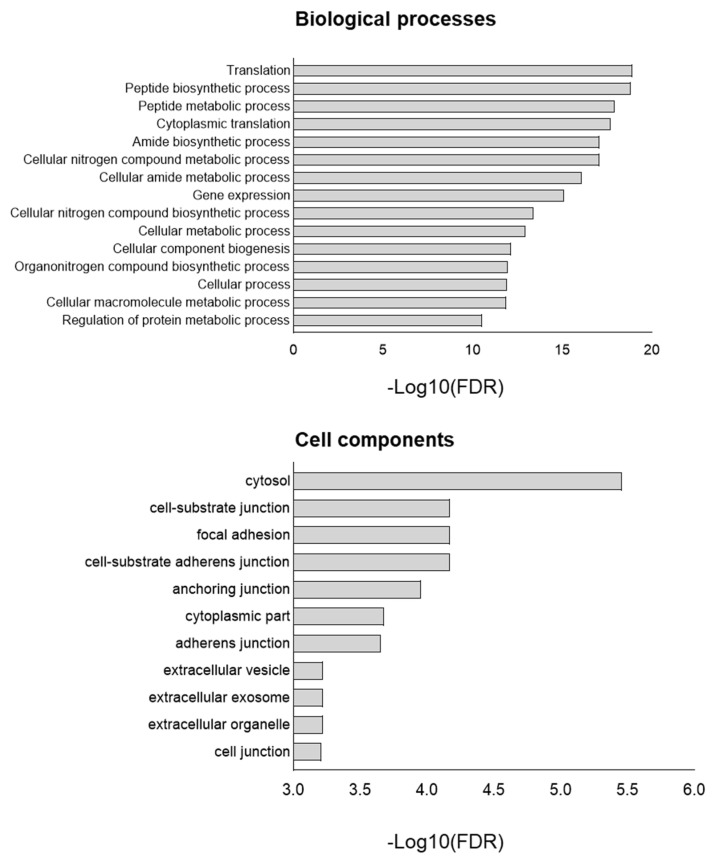
GO of proteins upregulated in *TP53*-KO cells compared to WT. −Log10(FDR)—−log10 false discovery rate.

Enrichment analysis (Gene Ontology terms) was employed to assess the impact of *TP53* knockout at the proteome level (Figure 3). The main differences were associated with such biological processes as translation, peptide biosynthetic process, peptide metabolic process, cytoplasmic translation, and cellular nitrogen compound metabolic process. Cell component analysis revealed enrichment in the cytosol, cell–substrate junction, focal adhesion, anchoring junction, and cell–substrate anchoring junction in *TP53*-KO cells. In addition, we analyzed the data obtained from cells cultured in a monolayer for 72 h (Supplementary Table S1). Functional annotation of a total of 79 proteins with differential expression between WT and *TP53*-KO cells revealed enrichment in terms like ‘RNA binding’ (FDR = 1.08^−8^), ‘Cadherin binding’ (FDR = 2.57^−2^), and ‘Cell adhesion molecule binding’ (FDR = 2.48^−2^).

### 3.3. p53-Defficient HaCaT Cells Display the Features of EMT

**Figure 4:**
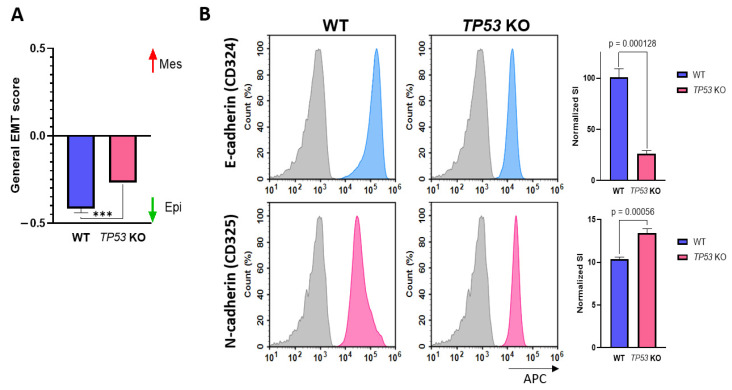
Verification of EMT in WT and *TP53*-KO HaCaT. (**A**) Evaluation of general EMT score in parental and *TP53*-KO HaCaT keratinocytes (−1—fully epithelial, +1—fully mesenchymal). Data are presented as mean ± SEM. ***—*p* value < 0.001. (**B**) Detection of E-cadherin and N-cadherin in flow cytometry analysis. Grey—unstained cells (isotypic control), blue—E-cadherin staining, pink—N-cadherin staining. Data are presented as mean ± SEM.

Differential expression analysis revealed that *TP53*-KO cells exhibited features of tumor cells and signs of EMT. Therefore, we conducted a general EMT score evaluation [26]. According to the results obtained, the general EMT score values for WT and *TP53*-KO HaCaT cells were −0.416 and −0.267, respectively (where −1 is fully epithelial and +1 is fully mesenchymal). These changes indicate that *TP53* knockout in HaCaT cells results in increased expression of EMT signature genes, whereas p53 appears to inhibit the implementation of this program (Figure 4A). To verify the results of the EMT score analysis, we assessed E-cadherin (CD324) and N-cadherin (CD325) expression by flow cytometry analysis (Figure 4B). E-cadherin expression was significantly higher in WT cells, whereas N-cadherin expression was higher in *TP53*-KO cells. Such changes in E-/N-cadherin expression are typical of cells undergoing EMT [57].

### 3.4. Knockout of TP53 Enhances Migration and Suppresses Epidermal Differentiation in HaCaT Cells

**Figure 5:**
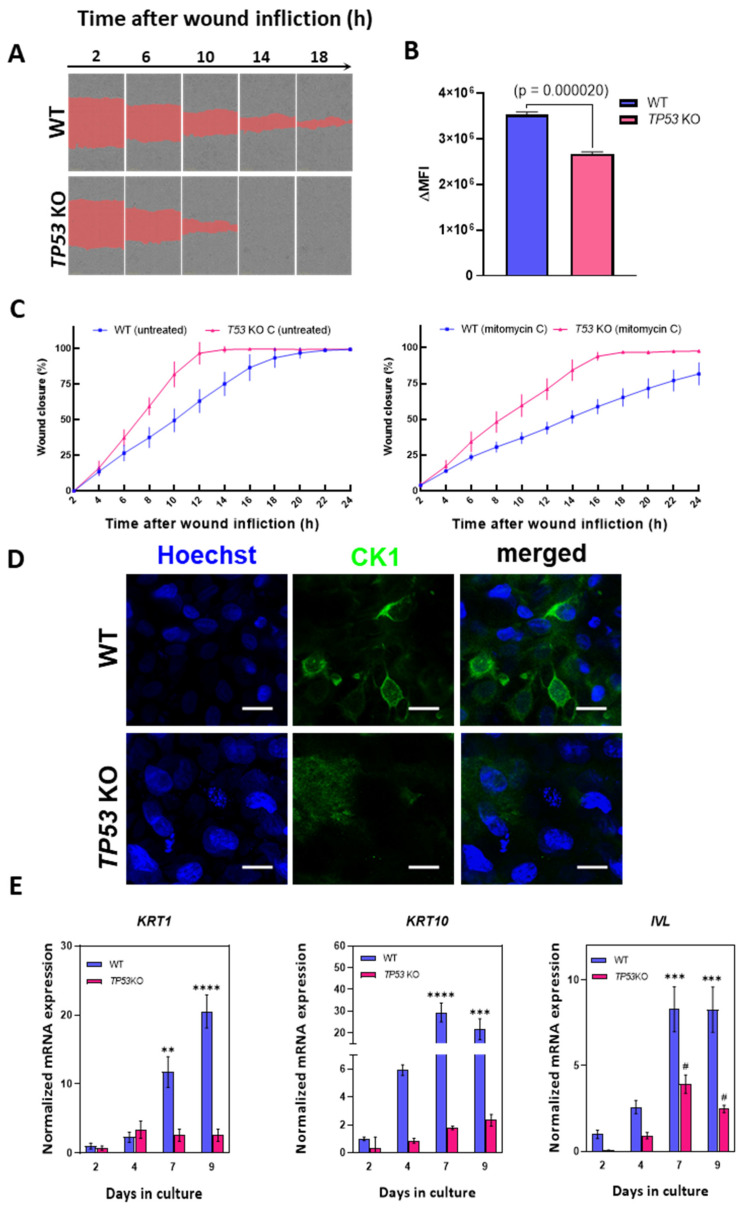
Analysis of migration and differentiation in parental and *TP53*-KO HaCaT keratinocytes (**A**) Scratch test: comparison of wound closure rates in WT and *TP53*-KO HaCaT (mitomycin C-treated). The images represent one of five technical replicates. (**B**) Analysis of the proliferation rate in WT and *TP53*-KO cells using Cytotrace^TM^ Red fluorescent probe. Data are presented as mean ± SEM. (**C**) The rates of wound closure by untreated (left) and mitomycin C-pretreated (right) WT and *TP53*-KO cells. Data are presented as mean ± SEM. (**D**) Keratin 1 (CK1) staining in WT and *TP53*-KO cells after 14 days of culture in cell culture inserts (air–liquid interface). Scale bar: 20 µm. (**E**) Analysis of epidermal differentiation markers in parental and *TP53*-KO HaCaT cells during culture in high-calcium medium. Data are presented as mean ± SEM. ****—*p* value < 0.0001; ***—*p* value < 0.001; **—*p* value < 0.005 compared to WT control (day 2); #—*p* value < 0.05 compared to *TP53*-KO control (day 2).

We postulated that the outcomes of omics data analysis in *TP53*-KO cells may indicate altered motility and migration rates compared to those in WT HaCaT cells. To test this hypothesis, we performed a migration assay (scratch test) to determine the effect of *TP53* inactivation on cell migration (Figure 5A,C). According to the scratch test results, *TP53*-KO cells filled the wound area faster than WT cells (Figure 5C). Thus, the wound closure times for WT and *TP53*-KO cells were 18–22 and 12–14 h, respectively. As proliferation may strongly affect wound closure rates, we measured proliferation rates in WT and *TP53*-KO cells using the Cytotrace^TM^ fluorescent probe (Figure 5B). A proliferation assay revealed that *TP53* inactivation results in decreased proliferation rates. Additionally, cells were pretreated with mitomycin C to suppress proliferation during wound closure, as previously reported [58,59]. As with untreated cells, *TP53*-KO cells filled the wound area faster than WT cells. After mitomycin C treatment, *TP53*-KO cells filled the lesion within 16–18 h, whereas WT cells filled the lesion in more than 24 h (Figure 5A,C, Video S1). Under both experimental conditions, the wound was closed as a result of collective migration (cohesive migration following leader cells). No individual cells colonizing the wounds were observed.

Proteomic and transcriptomic analyses revealed a significant decrease in involucrin (*IVL*) protein and mRNA expression. Involucrin is an important marker of epidermal differentiation of keratinocytes [60]; thus, we evaluated the expression of several epidermal differentiation markers during the induction of this program. Epidermal differentiation was induced by prolonged (9 days) cultivation in high-calcium medium (2.8 mM) as suggested by Wilson et al. [61].

*KRT1*, *KRT10*, and *IVL* were selected as target genes. These genes are expressed during keratinocyte differentiation and are considered key markers of advanced stages of differentiation [6,49,62]. Analysis of the mRNA level revealed an increase in the expression of all three markers in WT cells, but not in *TP53*-KO cells, in which this program was either completely suppressed or delayed (Figure 5E).

Culturing at the air–liquid interface induces epidermal differentiation [63]. We assessed keratin 1 (CK1) expression in cell cultures following a 14-day incubation period under air–liquid interface conditions, utilizing cell culture inserts to induce epidermal differentiation. ICC staining revealed that keratin 1 was only detected in the WT cells, which was consistent with the qPCR results (Figure 5D).

### 3.5. p53-Defficient HaCaT keratinocytes Possess Altered PD-L1 Expression

**Figure 6:**
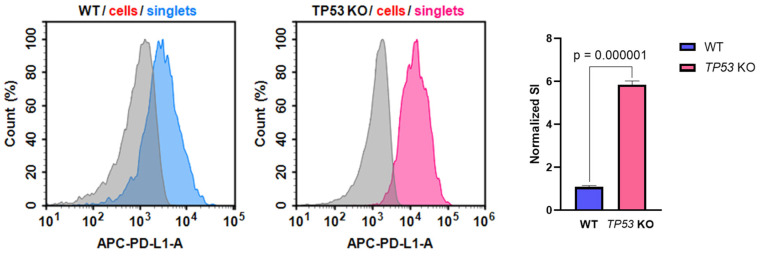
Expression of PD-L1 in WT and *TP53*-KO HaCaT cells. Grey—unstained cells (isotypic control), blue—PD-L1 staining in WT cells, pink—PD-L1 staining in *TP53* KO cells. Data are presented as mean ± SEM.

Altered *CD274* (PD-L1) expression in *TP53*-KO HaCaT cells is of particular interest in the context of keratinocyte biology and transformation. Thus, PD-L1 expression was verified at the protein level using flow cytometry analysis (Figure 6). The analysis revealed that the PD-L1 stain index (SI) was significantly higher in *TP53*-KO HaCaT cells than in WT cells.

## 4. Discussion

Given the extremely broad network of transcriptional activity of mutant p53 in HaCaT cells, we assessed the impact of p53 inactivation on HaCaT cells using transcriptomic and proteomic profiling. Proteomic profiling revealed that *TP53* knockout significantly affected translation, metabolic processes, and processes related to protein biosynthesis (Figure 3). Cell component analysis (Gene Ontology) revealed significant changes in proteins related to terms such as ‘cell-substrate junction’, ‘focal adhesion’, ‘cell-substate adherens junction’, ‘anchoring junction’, and others. At the transcriptomic level, processes associated with the activation of several signaling pathways, including PI3K, EGFR, NF-kB, and WNT, have garnered considerable interest (Figure 2). Some signaling pathways were downregulated (JAK-STAT, TGFB). In addition, several migration-associated genes such as *LCP-1*, *CD274*, *DLC-1*, *AREG,* and others were upregulated.

Based on the results of omics profiling, we hypothesized that p53 inactivation affects the migration rate of HaCaT cells. We employed a wound-healing assay (scratch test) to show that *TP53*-KO HaCaT cells had an increased rate of wound closure compared to WT cells. Additionally, we pretreated cells with mitomycin C to rule out the influence of proliferation on wound closure (Figure 5A,C). As with untreated cells, the wound closure rate was significantly higher in *TP53*-KO cells. The decreased proliferation rate of *TP53*-KO cells, compared to WT cells, was shown further using the Cytotrace^TM^ fluorescent probe (Figure 5B). Thus, we established that p53 inactivation results in altered migratory activity of WT and *TP53*-KO HaCaT cells. Notably, collective migration is typical for HaCaT cells. During this type of migration, cells cohesively migrate following the leader cells, whereas individual cells colonizing the wound may not be observed [58,59]. HaCaT keratinocytes retain the ability to implement the epidermal differentiation program [5,6,49]. Moreover, it has been shown that p53 is involved in the regulation of HaCaT cell differentiation [64]. We report that involucrin expression was significantly higher in wild-type cells than in *TP53*-KO HaCaT cells. In addition, the expression of differentiation markers, such as *KRT1*, *KRT10*, and *IVL*, was markedly reduced in *TP53*-knockout cells upon the induction of differentiation (Figure 5D,E). Notably, the inactivation of p53 in primary keratinocytes has very different consequences. Freije et al. showed that *TP53* knockdown leads to cell death by inducing squamous differentiation [65]. However, we did not observe a trend toward spontaneous cell death or induction of differentiation in *TP53*-KO HaCaT cells.

An increased migration rate and impaired differentiation are important hallmarks of the EMT. Transcriptomic profiling revealed several EMT features in *TP53*-KO HaCaT cells, including the upregulation of important EMT mediators, such as *CD274* and *AREG*. EMT score evaluation revealed that *TP53* inactivation resulted in a shift in the transcriptomic profile to a mesenchymal-like phenotype (Figure 4A). Additionally, we confirmed these results by measuring E-/N-cadherin expression in WT and p53-deficient cells (Figure 4B). Overall, the results clearly indicated signs of EMT in *TP53*-KO HaCaT cells. Notably, the observed collective migration does not contradict the implementation of the EMT program, since this migration type is common for cells with a partial EMT phenotype [66,67].

Altered *CD274* (PD-L1) expression was observed in *TP53*-KO HaCaT cells (Figure 6). In normal keratinocytes, PD-L1 expression is maintained at a low level, whereas overexpression of PD-L1 significantly characterizes tumors including cSCC and melanoma [68,69]. In melanomas, altered PD-L1 expression is frequently observed in malignant melanocytes and immune cells in the tumor microenvironment [70]. This is consistent with the altered migratory activity and transcriptomic alterations, as altered PD-L1 expression is largely characteristic of tumor cells. These findings, along with the results of omics profiling, indicate that *TP53*-KO cells possess a pro-oncogenic phenotype.

The role of p53, especially its mutant forms, in tumor progression is unclear. HaCaT cells provide a convenient model for studying UV signature mutations in *TP53* and their effects on various processes in human keratinocytes. At the same time, HaCaT cells are considered normal keratinocytes, while mutant p53 in HaCaT cells is referred to as nonfunctional or defective. CRISPR-mediated *TP53* knockout leads to significant changes at the transcriptomic level. The core proteomic differences between wild-type and *TP53*-KO HaCaT cells manifested as altered translation, protein biosynthesis, and cell adhesion. In addition, p53-deficient HaCaT cells are characterized by increased migration rates and reduced epidermal differentiation capacity, displaying signs of EMT and a pro-oncogenic phenotype.

The oncosuppressive functions of p53 are not limited by its antiproliferative activity but extend to other processes, including the regulation of migration. The impact of p53 on cell motility is largely manifested through actin cytoskeleton regulation via Rho signaling [71,72]. P53 also maintains a transcriptional program, preventing the EMT. Loss of this program in p53-null tumors results in the expression of an EMT-like phenotype [73]. Furthermore, p53 suppresses the EMT by regulating non-coding miRNAs of the miR200 family [74,75]. Our results are in line with these data, as *TP53* knockout results in altered migration and other features of the EMT. The implementation of pro-tumor programs in p53-deficient HaCaT cells may indicate that, despite the presence of mutations in *TP53*, the encoded protein retains its tumor suppressor functions.

## Figures and Tables

**Table 1:** Primers used in this study.

Gene	Forward Primer	Reverse Primer
*GAPDH*	5′- TCGACAGTCAGCCGCATCTTCTTT -3′	5′- ACCAAATCCGTTGACTCCGACCTT -3′
*ACTB*	5′- TCAGAAGGATTCCTATGTGGGCGA -3′	5′- CACGCAGCTCATTGTAGAAGGTGT -3′
*KRT10*	5′- AGCATGGCAACTCACATCA -3′	5′- GTCGATCTGAAGCAGGATGTT -3′
*KRT1*	5′- GCGGACAAATGCAGAGAATG -3′	5′- TGCTTGGTAGAGTGCTGTAAG -3′
*IVL*	5′- 5′-CCAAAGCCTCTGCCTCAG -3′	5′- GTATTGACTGGAGGAGGAACAG -3′

## Data Availability

Raw transcriptome data files are publicly available on the NCBI SRA (https://www.ncbi.nlm.nih.gov, accessed on 14 August 2023). The submission number is PRJNA1005459 for both WT and *TP53*-KO cells.

## Supplementary Materials

The following supporting information can be downloaded at https://www.mdpi.com/article/10.3390/cimb46020094/s1; Table S1: Differences between WT and *TP53*-KO cells at transcriptome and proteome levels. Video S1: Wound-healing assay.
